# Prevalence of Autoimmune Diseases in Individuals Living with HIV in Korea: A Nationwide Population-Based Study

**DOI:** 10.3390/medicina62061178

**Published:** 2026-06-17

**Authors:** Dongwoo Kim, Hongdeok Seok, Jae Hyun Jung

**Affiliations:** 1 Department of Internal Medicine, Korea University College of Medicine, Seoul 02841, Republic of Korea; 2 Division of Gastroenterology and Hepatology, Department of Internal Medicine, Korea University Ansan Hospital, Ansan 15355, Republic of Korea; 3 Department of Occupational and Environmental Medicine, Busan Adventist Hospital, Sahmyook Medical Center, Busan 49230, Republic of Korea; 4 Division of Rheumatology, Department of Internal Medicine, Korea University Ansan Hospital, Ansan 15355, Republic of Korea

**Keywords:** HIV infection, autoimmune diseases, prevalence, epidemiology

## Abstract

*Background and Objectives*: Human immunodeficiency virus (HIV) infection is associated with immune dysregulation, which may influence the development of autoimmune diseases. However, population-based evidence on the prevalence of autoimmune diseases in individuals living with HIV remains limited, particularly in Asian populations. This study aimed to evaluate the prevalence of autoimmune diseases in individuals living with HIV in Korea using nationwide population-based data. *Materials and Methods*: We conducted a cross-sectional analysis using the Health Insurance Review and Assessment Service National Patient Samples from 2012 to 2015, including 4,851,064 individuals aged ≥15 years. HIV infection and autoimmune diseases were identified using ICD-10 codes. The prevalence of autoimmune diseases in individuals with HIV infection was compared with that in the general population. Antiretroviral therapy (ART) status was determined based on prescription records. *Results*: A total of 1023 individuals were identified with HIV infection, all of whom were receiving antiretroviral therapy. The overall prevalence of autoimmune diseases was 4.4% in males and 3.6% in females with HIV, without significant differences compared to controls. However, the prevalence of ulcerative colitis in males (*p* = 0.030) and of dermatomyositis in females (*p* = 0.011) was higher in individuals with HIV. *Conclusions*: Although the overall prevalence of autoimmune diseases was not significantly increased in individuals living with HIV, certain autoimmune diseases—particularly ulcerative colitis in men and dermatomyositis in women—showed a higher prevalence. As these findings were based on small case numbers, they should be approached with caution. The results are best regarded as hypothesis-generative observations that warrant further investigation rather than findings on which clinical practice should currently be based. Further research using large datasets is warranted to confirm these associations and clarify the underlying immunological mechanisms.

## 1. Introduction

Human immunodeficiency virus (HIV) infection leads to immune dysregulation, primarily through depletion of CD4+ T cells [1]. HIV infection is associated with alterations in immune homeostasis and lymphocyte subsets, including changes in CD4+ and CD8+ T-cell populations, as well as B cells and natural killer cells. In addition to abnormal numbers of lymphocytes, dysregulation of B and T cells and polyclonal B-cell activation occurs [2]. Chronic immune activation and cytokine imbalance contribute to immune dysfunction and may play a role in the development of autoimmune diseases [2,3]. Differentiation from autoimmune diseases may be more difficult in these patients than in the general population due to increased positivity of autoantibodies, including rheumatoid factor, anti-cyclic citrullinated peptide antibody, and antinuclear antibody, and similar symptoms such as rheumatoid-like arthritis in individuals living with HIV [3,4]. However, the association between HIV infection and specific autoimmune diseases remains unclear.

Previous studies have reported an increased incidence of certain autoimmune diseases in individuals living with HIV, although findings vary according to antiretroviral therapy status [5,6]. Immune system abnormalities are associated with HIV infection and may be partially restored by ART. ART may influence the development and progression of autoimmune diseases. The number of HIV infections continues to increase in Korea. Moreover, more than 90% of individuals living with HIV are receiving ART, which is provided free of charge [7]. In addition to epidemiological and socioeconomic factors, genetic factors may also influence the development of HIV infection and autoimmune diseases. The prevalence and spectrum of autoimmune diseases in Korea may differ from those reported in other countries [8]. Therefore, the prevalence of autoimmune diseases in individuals living with HIV may differ in Korea.

HIV infection is associated with progressive depletion of CD4+ T lymphocytes and broad immune dysregulation, including chronic immune activation, polyclonal B-cell activation, disruption of regulatory T-cell function, and persistent inflammation that may persist even under effective antiretroviral therapy [1]. These immunological perturbations create a complex environment in which autoimmune phenomena may either be suppressed by progressive immunodeficiency or, conversely, be triggered by immune reconstitution, molecular mimicry, or chronic inflammation. The clinical consequences of these competing mechanisms remain incompletely understood, and the resulting spectrum of autoimmune disease in people living with HIV has been the subject of growing interest [2,3].

Previous studies have examined the prevalence of autoimmune diseases in people living with HIV [2,4,5], but most have been conducted in single-center or cohort settings, in Western populations, or in the era preceding the widespread adoption of combination antiretroviral therapy. Few studies have used nationwide, population-based data, and even fewer have addressed Asian populations [6], where the genetic background and epidemiology of both HIV infection and autoimmune diseases differ from Western settings. Korea, in particular, has a low HIV prevalence and a well-organized national health insurance system that provides comprehensive coverage of clinical encounters and prescriptions, offering a valuable opportunity to examine the co-occurrence of HIV infection and autoimmune diseases at the population level.

The use of steroids and immunosuppressive agents can affect the progression and treatment of HIV infection [4]. Cardiovascular diseases, renal diseases, bone disorders, and cancers are more common in those living with HIV than in the general population, which overlaps with the comorbidities observed in some autoimmune diseases [9,10]. However, population-based data in Asian populations remain limited. Therefore, investigating the prevalence of autoimmune diseases in individuals living with HIV may provide clinically relevant information for differential diagnosis and management. In this study, we aimed to investigate the prevalence of autoimmune diseases in individuals living with HIV in Korea and to compare these findings with those in the general population.

## 2. Materials and Methods

### 2.1. Data Source and Study Subjects

Data were obtained from the National Patient Samples of the Health Insurance Review and Assessment Service (HIRA) from January 2012 to December 2015 (dataset numbers: HIRA-NPS-2012-0133, HIRA-NPS-2013-0143, HIRA-NPS-2014-0152, and HIRA-NPS-2015-0151). HIRA is a nationwide claims database operated by the Korean government. HIRA provides various medical information, including basic information such as patient age and sex, disease information based on diagnostic codes, and medication information through drug codes [11]. HIRA-NPS includes approximately 3% of the total population using a stratified sampling method. The stratification variables were sex and age, with age categorized into 5-year intervals. A stratified probability sampling method with 32 layers was applied [12]. There were a total of 5,755,633 individuals in the 2012–2015 HIRA-NPS dataset. Of these, 4,851,064 individuals aged ≥15 years were included in the analysis (2,332,623 males and 2,518,441 females). The HIRA-NPS is a 3% stratified random sample of the entire Korean population enrolled in the National Health Insurance Service, which provides near-universal coverage. The sample is stratified by age, sex, and insurance type to be nationally representative, and it includes information on diagnoses (using KCD-7 codes, the Korean adaptation of ICD-10), prescriptions, procedures, and demographic characteristics.

### 2.2. Definition of HIV Infection and Autoimmune Diseases

The diagnostic codes of HIV infection were identified using B20–24 of the Korean Classification of Disease, 7th edition (KCD-7), which is the Korean version of the International Classification of Disease, 10th revision (ICD-10). Patients with HIV infection were defined as those with the relevant diagnostic codes recorded as either a primary or secondary diagnosis, and the control group was defined as those without these diagnostic codes. The autoimmune diseases and diagnostic codes included in this study were as follows: RA, M05, M06.0, and M06.2–9; AS, M45; SLE, M32; systemic sclerosis, M34; polymyositis, M33.2; dermatomyositis, M33.1 and M33.9; antiphospholipid syndrome, D68.6B; Sjögren’s syndrome, M35.0A–D; Behçet’s disease, M35.20–22, 28 and 29, and N77.8A; vasculitis except Behçet’s disease, M30.0–9; ulcerative colitis, K51.0, 2, 3, 8, and 9, and M07.50–58; Crohn’s disease, K50.00–02, 10–12, 19, 80–82, 89–92, and 99; sarcoidosis, D86.0–3, 8, and 9; psoriasis, L40.00, 08, 1, 4, 5, 8, and 9; Graves’ disease, E05.0–4, and 8; Hashimoto’s thyroiditis, E06.3A; autoimmune hepatitis, K75.4; and primary biliary cholangitis, K74.30–32 and 39. Individuals with an autoimmune disease diagnostic code as a primary or secondary diagnosis were defined as patients with an autoimmune disease; otherwise, they were classified as not having an autoimmune disease. To improve the specificity of disease ascertainment, each autoimmune disease was defined as the presence of the corresponding KCD-7 code as the principal or secondary diagnosis on at least two separate outpatient visits, or on one inpatient admission, during the study period. This operational definition has been widely used in prior claims-based studies of autoimmune diseases in Korea [8,13] to reduce misclassification arising from rule-out diagnoses.

### 2.3. Antiretroviral Therapy

ART status was defined based on the prescription of one or more antiretroviral drugs, including abacavir, adefovir, amprenavir, atazanavir, bictegravir, clevudine, cobicistat, darunavir, delavirdine, didanosine, dolutegravir, doravirine, efavirenz, elvitegravir, emtricitabine, enfuvirtide, entecavir, etravirine, fosamprenavir, indinavir, lamivudine, lopinavir, nelfinavir, nevirapine, raltegravir, rilpivirine, ritonavir, saquinavir, stavudine, telbivudine, tenofovir, tipranavir, zalcitabine, and zidovudine. The corresponding drug codes were J05AE01–10, J05AF01–13, J05AG01–06, J05AR01–27, and J05AX07, 08, and 12. Because HIV infection was identified using the diagnostic codes (KCD-7 B20–24), the prescription of these drugs was used only to determine ART status within the HIV-defined population.

### 2.4. Statistical Analysis

The prevalence of each autoimmune disease in people with HIV infection and in control subjects was compared using Fisher’s exact test, and a *p* < 0.05 was considered statistically significant. Analyses were stratified by sex. All statistical analyses were performed using SAS Viya 4 (SAS Institute Inc., Cary, NC, USA). Continuous variables are presented as numbers and percentages, with percentages reported to one decimal place; for prevalences below 0.1%, two decimal places are shown to preserve the resolution of small but non-zero values. Prevalence is expressed per 1000 individuals (‰) to facilitate the comparison of very low prevalences between groups. For the two associations that remained statistically significant, prevalence ratios with 95% confidence intervals were calculated using the natural log transformation of the prevalence ratio, with the standard error derived from the delta method.

## 3. Results

### 3.1. Prevalence of HIV Infection and Autoimmune Diseases

There were 939 males and 84 females with HIV infection, for a total of 1023 individuals, and the overall prevalence of HIV infection was 0.021% (0.040% in males and 0.003% in females). All people with HIV infection included in this study were receiving one or more antiretroviral drugs. Among the autoimmune diseases included in the study, RA had the highest prevalence at 2.4% (1.6% in males and 3.1% in females), followed by psoriasis (0.7% overall, 0.8% in males and 0.5% in females), Graves’ disease (0.5% overall, 0.3% in males and 0.7% in females), and Hashimoto’s thyroiditis (0.4% overall, 0.1% in males and 0.7% in females).

### 3.2. Prevalence of Autoimmune Diseases in People with HIV Infection

The overall prevalence of autoimmune disease in people with HIV infection was 4.4% in males, higher than in those without HIV infection, and 3.6% in females, lower than in those without HIV infection. However, these differences were not statistically significant. Table 1 presents the prevalence of each autoimmune disease in patients with and without HIV infection. The prevalence of ulcerative colitis in males (4/939; prevalence ratio [PR] 3.45, 95% CI 1.30–9.17; *p* = 0.030) and of dermatomyositis in females (1/84; PR 90.58, 95% CI 12.87–637.46; *p* = 0.011) was higher in individuals with HIV infection. The wide confidence intervals, reflecting the very small number of cases, indicate that these estimates are statistically fragile.

Prevalence is expressed per 1000 individuals (‰). Although a *p* value of <0.05 was used as the threshold for statistical significance, differences based on single-digit case numbers were interpreted with caution and are not described as statistically significant.

## 4. Discussion

This nationwide population-based study provides clinically relevant evidence on the prevalence of autoimmune diseases in individuals living with HIV in Korea. The overall prevalence of autoimmune diseases in individuals with HIV infection receiving ART did not differ significantly from that in individuals without HIV infection. However, ulcerative colitis in men and dermatomyositis in women showed a higher prevalence in individuals with HIV infection. Since these findings were based on very small numbers of cases, they were not considered to represent statistically robust associations and should be interpreted with caution; nonetheless, they may suggest a potential link between HIV infection and these conditions that warrants confirmation in larger studies.

Several mechanisms have been proposed to explain the potential co-occurrence of HIV infection and autoimmune disease [2,3]. Persistent HIV-related immune activation, gut microbial translocation, and chronic inflammation may contribute to the development of organ-specific autoimmunity, even when peripheral CD4+ T-cell counts are restored by antiretroviral therapy [14]. In addition, immune reconstitution following ART initiation has been reported to unmask or precipitate autoimmune phenomena in some individuals [3]. These mechanisms provide a plausible biological context for the observations reported here, while underscoring that any associations must be interpreted in light of the very small case numbers and the methodological limitations of administrative data.

Gut-associated lymphoid tissues (GALTs) are a critical site of CD4+ T-lymphocyte depletion and play an important role in HIV replication and the development of inflammatory bowel disease, such as ulcerative colitis [15,16]. HIV infection leads to a decrease in CD4+ T cells in the intestinal tract, resulting in disruption of the gastrointestinal mucosa, dysbiosis, and chronic inflammation similar to that observed in inflammatory bowel disease. In addition, diarrhea is a common symptom in people with HIV infection and ulcerative colitis [17]; thus, HIV-associated colitis and ulcerative colitis may be clinically difficult to distinguish. However, these diseases can be differentiated, as HIV-associated colitis is characterized by less mucosal damage or ulceration and distinct sites of inflammation [14]. ART increases the number of CD4+ T cells and may attenuate ulcerative colitis, whereas ulcerative colitis may worsen in uncontrolled HIV infection [18] and can be fatal due to intestinal bleeding or perforation [19]. Thus, determining the coexistence of HIV infection and ulcerative colitis is important for appropriate management.

Dermatomyositis is an inflammatory myopathy characterized by skin lesions and muscle weakness and is more common in women [13]. Dermatomyositis is mediated by CD4+ T cells and B lymphocytes. The cell-mediated response is led by perifascicular CD8+ T cells and macrophages that invade myocytes expressing major histocompatibility complex (MHC) class I antigens. HIV infection leads to depletion of CD4+ T cells and an inversion of the CD4+/CD8+ T-cell ratio. It may also trigger autoantibody production associated with dermatomyositis [20]. The mechanism underlying the relationship between HIV infection and dermatomyositis remains unclear, and only a few cases of co-occurrence have been reported. However, in people with HIV infection, especially women presenting with skin involvement and muscle weakness, dermatomyositis should be considered in the differential diagnosis.

HIV infection mainly destroys CD4+ T cells involved in cellular immunity. Although HIV infection has clinical manifestations caused by the virus, secondary diseases also occur due to decreased immune function. Acquired immune deficiency syndrome (AIDS) involves various complications, such as infectious diseases or malignant tumors, which occur as immunity decreases. ART is typically initiated when AIDS is diagnosed or when the CD4+ T-cell count is low, even in the absence of symptoms [21]. HIV activity and ART are closely related to the function and number of CD4+ T cells, which may influence the development of autoimmune diseases. All people with HIV infection included in this study were receiving ART. The ART regimens used in this study may differ from those reported in other countries [5,6].

Since this study analyzed nearly 5 million individuals, it provides a representative estimate of the prevalence of autoimmune diseases in people with HIV infection in Korea. In addition, since individuals living with HIV are systematically managed through a national healthcare system, the diagnosis of comorbidities may be relatively reliable. The strengths of this study include the use of a large, nationally representative sample, the inclusion of a wide range of autoimmune diseases, and stratification by sex, which allowed us to detect sex-specific patterns that might be obscured in pooled analyses. The HIRA-NPS provides comprehensive coverage of inpatient and outpatient encounters across the Korean population and is therefore well-suited to estimating the prevalence of relatively rare comorbidities. To our knowledge, this is the first nationwide, population-based estimate of the prevalence of multiple autoimmune diseases in people living with HIV in Korea, and one of the few such estimates from an Asian population. However, this study has some limitations. First, the cross-sectional design precludes causal inference regarding the relationship between HIV infection and the development of autoimmune diseases. Second, diagnoses of HIV and autoimmune diseases based on administrative claims data recorded by physicians or hospitals may be less accurate than those made in a prospective clinical setting. Third, the same systematic management that improves diagnostic reliability may also introduce surveillance bias: as individuals living with HIV undergo more frequent clinical and laboratory evaluations than the general population, comorbid autoimmune diseases are more likely to be detected in this group, which may inflate the apparent prevalence relative to controls. More frequent monitoring of people with HIV can by itself produce an apparent excess of any detected comorbidity, and the higher prevalences of ulcerative colitis and dermatomyositis observed here may, at least in part, reflect this differential ascertainment rather than a true biological association. Fourth, as a population-based prevalence study, each autoimmune disease was examined as a separate, pre-specified outcome rather than as part of a single family of hypotheses, and the analysis was descriptive in nature. Given the very small number of cases in the HIV group, a conservative correction for multiple comparisons (e.g., Bonferroni) would have been overly stringent, substantially increasing the risk of type II error and obscuring potentially meaningful signals; the reported *p*-values were therefore not adjusted for multiple comparisons. Consequently, some of the elevated prevalences may represent chance findings, and the significant associations should be interpreted with appropriate caution. Fifth, the study period (2012–2015) predates the widespread adoption of several contemporary first-line ART regimens, such as integrase strand transfer inhibitor-based regimens. As ART composition influences immune reconstitution and may modulate the development of autoimmune phenomena, our findings may not fully reflect the current treatment landscape, and confirmation using more recent data is warranted. Finally, as the number of people with HIV infection was much smaller than that of people without HIV infection, the prevalence estimates for individual diseases are statistically fragile. In particular, dermatomyositis in women was observed in only one person with HIV infection; such findings are highly sensitive to single cases and must be interpreted with great caution.

## 5. Conclusions

In this nationwide study, the overall prevalence of autoimmune diseases did not differ significantly between people with and without HIV infection. A few conditions—most notably ulcerative colitis in men and dermatomyositis in women—showed a higher prevalence in people with HIV infection. However, these findings were based on very small case numbers and should be regarded as hypothesis-generating observations rather than established associations or a basis for current clinical practice. As the number of people with HIV infection continues to increase in Korea, further research using larger and more recent datasets is needed to determine whether these observations reflect genuine associations.

## Figures and Tables

**Table 1:** The prevalence of autoimmune disease in men and women with HIV infection in Korea.

	Men					Women				
	HIV Infection (*n* = 939)		Non-HIV Infection (*n* = 2,332,623)		*p*-Value	HIV Infection (*n* = 84)		Non-HIV Infection (*n* = 2,518,441)		*p*-Value
	*n*	‰	*n*	‰		*n*	‰	*n*	‰	
Rheumatoid arthritis	10	10.6	36,985	15.9	0.239	2	23.8	78,857	31.3	1.000
Ankylosing spondylitis	2	2.1	5621	2.4	1.000	0	0.0	4084	1.6	1.000
Systemic lupus erythematosus	1	1.1	1412	0.6	0.434	0	0.0	4925	2.0	1.000
Systemic sclerosis	0	0.0	90	0.0	1.000	0	0.0	478	0.2	1.000
Polymyositis	0	0.0	247	0.1	1.000	0	0.0	405	0.2	1.000
Dermatomyositis	0	0.0	206	0.1	1.000	1	11.9	331	0.1	0.011
Antiphospholipid syndrome	0	0.0	159	0.1	1.000	0	0.0	319	0.1	1.000
Sjögren’s syndrome	2	2.1	1665	0.7	0.146	0	0.0	4645	1.8	1.000
Behçet’s disease	2	2.1	1158	0.5	0.080	0	0.0	2472	1.0	1.000
Vasculitis (except Behçet’s disease)	0	0.0	376	0.2	1.000	0	0.0	617	0.2	1.000
Ulcerative colitis	4	4.3	2883	1.2	0.030	0	0.0	2372	0.9	1.000
Crohn’s disease	2	2.1	1697	0.7	0.150	0	0.0	1072	0.4	1.000
Sarcoidosis	0	0.0	0	0.0	1.000	0	0.0	222	0.1	1.000
Psoriasis	12	12.8	18,198	7.8	0.091	0	0.0	13,717	5.4	1.000
Graves’ disease	3	3.2	6479	2.8	0.749	0	0.0	16,289	6.5	1.000
Hashimoto’s thyroiditis	1	1.1	2741	1.2	1.000	0	0.0	16,668	6.6	1.000
Autoimmune hepatitis	1	1.1	655	0.3	0.232	0	0.0	1316	0.5	1.000
Primary biliary cholangitis	1	1.1	247	0.1	0.095	0	0.0	456	0.2	1.000
Total	41	43.7	80,819	34.6	0.129	3	35.7	149,245	59.3	0.490

## Data Availability

The data used in this study are available from the Health Insurance Review and Assessment Service (HIRA), but restrictions apply to the availability of these data due to privacy regulations, and thus they are not publicly available.

## Abbreviations

The following abbreviations are used in this manuscript:

HIV	Human immunodeficiency virus
ART	Antiretroviral therapy
AIDS	Acquired immune deficiency syndrome
RA	Rheumatoid arthritis
